# Assessing musculoskeletal injury risk and skeletal changes from backstrap loom weaving and traditional embroidery in Chiapas, Mexico

**DOI:** 10.1371/journal.pgph.0004574

**Published:** 2025-04-29

**Authors:** Alizé Lacoste Jeanson, Monserrat Romero Morales, Rosa Itzel Flores Luna

**Affiliations:** 1 Instituto de Investigaciones Antropológicas, Universidad Nacional Autónoma de México, Coyoacán, Ciudad de México, México; 2 UMR 5199 PACEA (De la Préhistoire à l’Actuel: Culture, Environnement et Anthropologie), Université de Bordeaux, Talence, France; 3 Facultad de Ingeniería, Universidad Nacional Autónoma de México, Coyoacán, Ciudad de México, México; PLOS: Public Library of Science, UNITED STATES OF AMERICA

## Abstract

Few medical studies are led in places where social security services are almost inexistent, leaving a gap in knowledge about occupational health risks tied to traditional crafts. This study investigates how traditional textile work—specifically embroidery and backstrap loom weaving work—affects the body in the Highlands of Chiapas, where these crafts represent a substantial part of thousands of women daily activity. Using multi-angle video recordings and interviews with adult women skilled in these crafts, the study evaluates musculoskeletal injury risk through biomechanical analysis. It examines movement types, repetition, involved body parts and muscles, and static postures. Tools such as the Rapid Entire Body Assessment (REBA), Standardized Nordic questionnaires, and evaluation of skeletal changes support this assessment. Findings show frequent, repetitive motions in the upper limbs and fingers, often approaching joint mobility limits (e.g., elbows flexed 60–100°, wrists >15°). These are combined with static, physically demanding postures—spine, neck, and legs are under constant strain due to ground-level sitting positions with the hips flexed at 90°, neck >20°, and knees deeply flexed in some cases (cross-legged or kneeling). Potential musculoskeletal injuries include tendinitis, carpal tunnel syndrome, tenosynovitis, bursitis, spinal disc herniation, and spondylolysis. Skeletal changes would mainly affect the hands, wrists, elbows, and spine, with asymmetry in embroidery and more symmetry in backstrap weaving. These may appear as localized entheseal changes and osteoarthritis. The study demonstrates the need of setting out preventive actions to reduce the injuries risk implied by traditional embroidery and backstrap loom weaving crafts. In order to assess actual musculoskeletal changes linked to those activities, a project is underway to examine bone markers specific to textile craftsmanship in ancient peoples of the same Maya area found buried with textile-making tools.

## Introduction

Textile craftsmanship is a deeply rooted cultural and economic activity with significant historical and contemporary relevance. In the Highlands of Chiapas, Mexico, the traditional practice of backstrap loom weaving and embroidery has existed for centuries, with archaeological and iconographic evidence tracing its origins back to at least the Classic Maya period (AD 200–900). These crafts played a significant role in local economies, particularly as fabrics were often used as tribute to state-like entities [1]. Despite the evidence of textile production in ancient Mesoamerica, little is known about the time investment, physical demands, or presence of craft specialists in pre-Hispanic societies.

In modern Chiapas, textile craftsmanship, although very ancient and traditionally practiced using minimal tools, has evolved in response to social and economic changes. Before the huge increase of tourism in 1994, backstrap loom weaving and traditional embroidery were primarily practiced for the household clothing production and local exchanges. Today, these activities contribute significantly to household economies, with thousands of women dedicating long hours to producing textiles for sale. In contrast, the foot-treadle loom, introduced during the Spanish invasion and predominantly used by men, was traditionally associated with larger-scale textile production. This gendered division of labor highlights the shifting cultural and occupational significance of textile production over time.

While the cultural and economic relevance of these crafts is well-documented, their physical demands and health implications remain underexplored in the region. The repetitive and physically strenuous nature of backstrap loom weaving and traditional embroidery often requires constrained postures—such as kneeling or sitting cross-legged on the ground—and repetitive upper-limb movements sustained over extended periods.

Research has demonstrated that industrial textile workers are highly susceptible to musculoskeletal disorders (MSDs) because of repetitive motions, postural constraints, and prolonged work durations. For example, studies conducted in textile factories across India, Turkey, and Thailand have consistently reported a high prevalence of MSDs among workers: Angeline and Bobby (2018) demonstrated significant work-related musculoskeletal risks among adolescent girls and young women employed in textile industries in Tamil Nadu, India at the level of neck and shoulder [2]; Berberoğlu and Tokuç (2013) found a high frequency of back, neck, and shoulder pain among workers in textile factories in Turkey [3]; and Keawduangdee et al. (2010) indicate that most of musculoskeletal pain is experienced at the level of the shoulder, lower back, neck and hand in workers of the textile industry in Khon Kaen, Thailand [4]. In a very general way Singh (2016) reports MSDs such as “carpal tunnel syndrome, forearm tendinitis, bicipital tendinitis, lower back pain, epicondylitis, neck pain, shoulder pain, and osteoarthritis of the knees” in clothing and textile industries works as the occupational diseases that have been observed [5].

While these studies offer valuable insights into the occupational risks faced by textile industry workers, they fail to capture the unique experiences of women artisans engaged in millennia-old textile crafts. Although some research has focused specifically on weavers [6], to the best of our knowledge, no studies have been conducted on backstrap loom weaving, or on traditional embroidery using a simple needle. It should also be noted that none of the studies mentioned above describes which specific activities, movements and static postures are adopted by clothing industry workers (merely referring to part of those activities as “sewing” in some cases), hence limiting full understandability of the actual risk—although some of them do make mention of mean hours of work per week. Industrial settings differ markedly from traditional textile production in terms of tools, time, work environment, and economic pressures. Backstrap loom weaving and embroidery, in contrast, are traditionally performed within informal and domestic spaces without access to ergonomic tools or occupational health resources. Moreover, the constrained postures inherent to these practices—combined with repetitive motions and limited rest—may exacerbate physical strain, risk of musculoskeletal disorders and long-term skeletal changes, yet remain largely unexamined in this specific cultural and occupational context.

In the specific context of indigenous textile workers in Chiapas, there is a notable gap in research on ergonomic risks and interventions. While the aforementioned studies emphasize the challenges faced by industrial textile workers, the lack of research on artisanal textile production prevents a comprehensive understanding of its health implications. Addressing this gap is critical, as indigenous artisans often face socioeconomic barriers to healthcare and occupational safety resources, exacerbating the long-term health impacts of their work.

Ergonomic principles provide a framework for understanding and mitigating these occupational risks by optimizing the interaction between workers, their tasks, and their work environment. Key principles such as maintaining neutral postures, minimizing repetitive loads, and integrating frequent micro-pauses can reduce physical strain and improve worker health [7]. Tools such as the Standardized Nordic Questionnaire [8] and the Rapid Entire Body Assessment (REBA) [9] are well-established methods for evaluating musculoskeletal symptoms and ergonomic risks, respectively, and have been effectively applied in various occupational settings. Kuorinka et al. (1987) developed the Standardized Nordic Questionnaire as a validated tool to identify MSD prevalence across occupations, while Hignett and McAtamney (2000) introduced REBA as a systematic method to assess postural risks in physically demanding tasks [8,9].

This study aims to assess the musculoskeletal risks and skeletal changes associated with backstrap loom weaving and traditional embroidery among women master artisans in the Highlands of Chiapas. A key component of this research is the application of ergonomic principles to analyze the physical demands of these activities and identify areas for potential intervention. By employing a detailed biomechanical analysis and validated ergonomic tools such as the standardize Nordic Questionnaire and REBA, this study systematically evaluates postures, movements, and the prevalence of musculoskeletal symptoms. The integration of ergonomic frameworks will provide critical insights into the relationship between constrained postures, repetitive motions, and the risk of MSDs, contributing to a broader understanding of occupational health in artisanal labor.

Ultimately, this research not only seeks to document the health impacts of traditional textile craftsmanship but also to promote culturally sensitive ergonomic interventions. By aligning occupational health considerations with the preservation of textile traditions, this study aims to enhance the well-being of women artisans while supporting the sustainability of an invaluable cultural practice.

## Methodology

### Ethics statement

A written informed consent was obtained for authorizing the investigator to record the information; it described the aim and methods of the investigation, its benefits, the use of the data and its protection, the insurance for participants of being able to freely leave the investigation and to ask for clarification. The data we collected are twofold: in the field, we recorded videos of participants while embroidering and backstrap loom weaving, and we performed Standardized Nordic questionnaires; back in the laboratory, we realized kinematic analysis of those activities, we applied REBA and analyze results of Standardized Nordic questionnaires in order to evaluate ergonomic risk of the postures. The data was collected between 28/10/2023 and 25/03/2024 and was analyzed up until May 2024. The investigation, recruitment methods, and informed consent, were approved by the Committee of Ethics of the Instituto de Investigaciones Antropológicas of the Universidad Nacional Autónoma de México.

### Characterization of study’s participants

The people analyzed as part of this case-study are exclusively women, as backstrap loom weaving and traditional embroidery are activities almost exclusive to this sex in Chiapas. They proceed from various towns of the Highlands of Chiapas, although most actually come from San Juan Chamula and identify as Tsotsil; one woman comes from San Juan Cancuc and identifies as Tseltal and almost all live in San Cristóbal de las Casas. The women are aged 33–53, none are obese or underweight, and they are all between 150 and 160 cm of height. The case-study being explorative, we obtained complete data for 5 and 4 women for embroidery and backstrap loom weaving, respectively. All the women participating in the study are masters in their crafts.

### Data collection procedures

In order to accurately understand the amplitude of the symptoms and the related changes of the skeleton to textile craftsmanship, we recorded videos of women while embroidering and weaving with five different angles: front, lateral (both sides), oblique and superior (head camera).

Standardized Nordic Questionnaires were used in order to assess musculoskeletal symptoms and their relationship to factors related to activities implied in textile production. Standardized Nordic questionnaires are based on nine anatomical regions and contain questions about the duration of the trouble (ache, pain or discomfort) and how much it is impeding [8]. It has been proven useful and reliable for use with a Mexican population [10].

### Analytical procedures

#### Biomechanical analysis.

Based on repetitive movements as well as static postures adopted for practicing the activity and the assessment of musculoskeletal symptoms, we estimated musculoskeletal injury risk and relative skeletal changes.

The kinematic analysis is complemented by a description of segment motions, joint actions, muscular implication and its relation to bones. More specifically, the study emphasizes the distal insertions of the muscles involved, as these sites reflect the angles and forces directly influencing movement. In the discussion section, a comparative analysis is undertaken, drawing parallels with sports and occupational activities that involve similar postures and/or movements, in order to further infer the potential changes of the skeleton related to traditional embroidery and backstrap loom weaving.

#### Musculoskeletal injury risk.

Musculoskeletal injury risk was evaluated through the analysis of ergonomic risk factor based on the recorded videos (evaluation of repetitive motions and identification of constrained postures) and through the analysis of the information collected through the Standardized Nordic questionnaires. The postural loading factor was then measured with the method called REBA [9]. REBA measures postural load exercised by constrained postures for each movement considering the following body parts: trunk, neck, legs, upper arms, lower arms, wrists. It is performed for each side (right and left). On top of considering the sum of postures implied in an activity, REBA adds an additional scoring based on:

- hand prehension type;
- whether loading (weight) is implied;
- whether postures are held for more than one minute;
- movements repeated more than four time per minute;
- existence of rapid large changes in postures and/or an unstable base.

The result is given by a score indicating occupational risk’s level that goes from negligible (score 1) to very high (score 15), corresponding to distinct level of ergonomic actions that would need to be taken in order to reduce risk level.

Occupational risk is defined by accidents or illnesses to which people are exposed while they are working or intended to work [7]. In case of an accident, the event (wound) occurs suddenly while in case of illness, the event develops gradually and roots from continued exposure. In Mexico, musculoskeletal disorders constitute the second cause of inability to work in workers affiliated to the IMSS (Mexican Social Security Institute) in 2022 [11]: spine injuries are the second case of working illnesses for women, after COVID-19, with a prevalence of 457 cases (16.6%); as for men, it is the second cause as well with 2358 occurrences (83.4%). Spine injuries’ most common risk factors are the constrained postures and repetitive motions [12,13]. The importance of the current paper resides in the fact that very little studies are made for quantifying and registering the exposure to such risks in populations who do not benefit from Social security services and/or who work as independent workers.

#### Skeletal changes.

Bone is a conjunctive tissue made of collagen fibers, glycoproteins and cells. The bone tissue mineralizes: its matrix stores mineral components, which tie up to the collagen fibers. Bone is an adaptive tissue, which responds to mechanical and biological constraints seeking for its homeostasis—as such it modifies its morphology. Bone functional adaptation serves for maintaining its structural and mechanical integrity through remodeling processes, which consist in addition, resorption, and substitution of bone material [14,15]. Bone functional adaptation occurs throughout life and modifies the morphology of bones both microscopically and macroscopically. It is determined both by the use of the muscular system and by biological events such as senescence, obesity, and other metabolic, nutritional and pathological factors. Although bones possess the ability to adapt to their biomechanical environment, this capacity can be surpassed under certain conditions. In such cases, bones may weaken because of a loss of mineral density or fracture as a result of structural damage. These instances reflect alteration rather than adaptation, although such alterations may remain asymptomatic. Therefore, rather than distinguishing between bone functional adaptation (physiological) or alteration (pathological), we will use the term ‘skeletal changes’ to describe modifications in bones’ morphology that may result from the activities of backstrap loom weaving and traditional embroidery.

It has notably been shown that in the human skeleton, activity or occupation-related skeletal changes are visible at the level of fibrocartilaginous enthesis, which are insertion sites of ligaments and tendons mostly at the joints [16–19]. This is congruent with data from occupational medicine and sport (e.g., [20,21]). Entheseal changes increase with age [22]. Since entheses acquire their ultimate appearance after the end of skeletal growth and development and seem to act as growth plates beforehand [19,23], they only are useful markers for activity occurring during adulthood.

Beyond entheseal changes, we consider alterations of joints and asymmetry of appendicular bones in order to get a fuller picture of skeletal changes related to handwoven textile techniques. We also provide comparisons with other occupations and selected sports-related conditions with comparable movements.

## Results

### Biomechanical analysis

**Fig 1:**
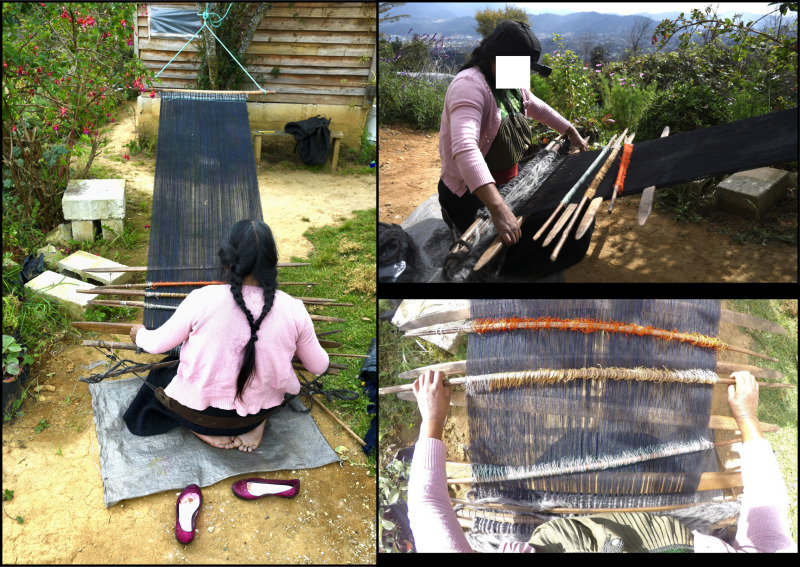
Catalina while backstrap loom weaving with wool (December 2023). Back, lateral right and superior views. Photo credits: Alizé Lacoste Jeanson.

**Fig 2:**
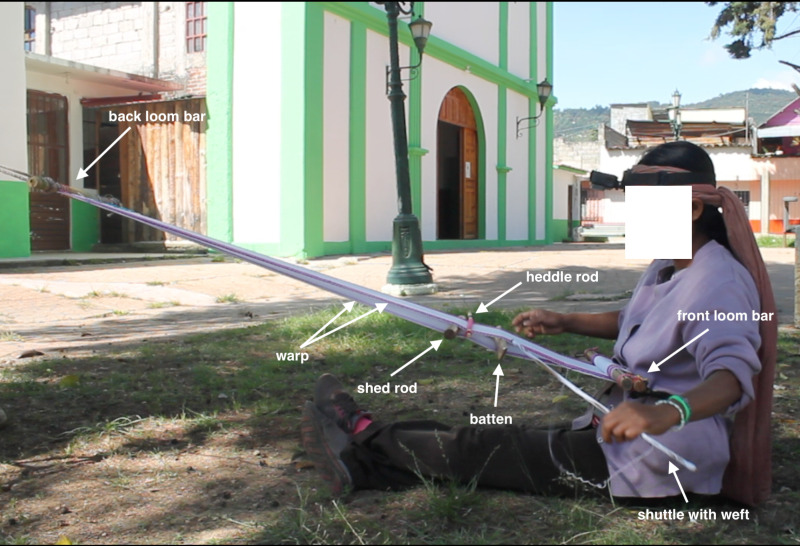
Juanita while backstrap loom weaving with cotton (November 2023). Tools’ names are identified on the picture. Photo credits: Alizé Lacoste Jeanson.

The traditional way of practicing both embroidery and backstrap loom weaving in Chiapas is with minimal tools and sitting on the ground. For traditional embroidering, no use is made of hoops; only a needle, a fabric and threads are required. Backstrap loom weaving is realized with the aid of a large handwoven backstrap harnessed on the women’s back at the level of the ilium (Fig 1), which connects the loom at the other end to a tree or a pillar and leaves it suspended in the air for working on it. The working area is almost always with similar dimensions, as the loom gets enrolled on itself as work progresses (Fig 1). Tools and loom’s parts are described in Fig 2. Tools size varies depending on the width of the loom, which ranges from 70 to 140 cm—the smallest ones are used to weave cotton and polyester; the largest ones are used to weave wool. The basic tools that are manipulated are battens [*machetes*], which are pieces of carved wood (either *hormigo* [*Platymiscium dimorphandrum*] or *guanacaste* [*Enterolobium cyclocarpum*]) measuring between 70 cm to 1.4 meter of length, used to divide the two layers of threads (warp [*urdimbre*])—for the smallest looms only a single small batten is used whereas the largest looms used to weave wool require the use of up to five large *machetes*, increasing considerably the weight of the worked loom and implying a different posture (the tallest one we weigh was 300 grams and three of those were used to work on the loom). A series of two or more wooden sticks are pulled and serve as dividers between the two layers of the loom (shed and heddle rods [*varas de paso y de lizo o alzadera*]). The thread (weft [*trama*]) is passed in between the warp with the aid of another wooden stick (shuttle [*bobina o lanzadera*]) around which said thread is tangled. The overall loom is maintained horizontally thanks to two main loom bars (back loom bar is up; front loom bar is down next to the body).

General body position for practicing both crafts is mostly constrained at the level of the lower limbs—the variant of backstrap loom weaving with wool being the only one which implies regular complete flexion of the lower limbs. For embroidering, the usual way of sitting is directly on the ground with either crossed or extended legs, or on a small chair (less than 30 cm high). Backstrap loom weaving is always practiced sitting on the ground—when the loom is not larger than the actual width of the hips, women sit directly on their buttocks with legs extended; when the loom is wider and heavier as for weaving wool, women sit on their heels with knees and dorsal portion of the feet resting on the ground (Fig 1). Those constrained body positions imply flexion (>20°) of the joints from cervical vertebrae to the lumbosacral joint and flexion of more than 90° of the coxofemoral joint, which is mostly permitted by the iliopsoas muscles—those insert distally on the femoral lesser trochanter. In cases where women sit cross-legged (traditional embroidery), the position is thus coupled with an abduction of the hip joint in which both muscles gluteus medius and gluteus minimus act upon (both insert distally on the femoral greater trochanter). The knee is flexed in cases when the person sits on a small chair, and hyperflexed when they sit with legs crossed or on their knees. Flexion of the knee is assisted by muscles gracilis, sartorius, popliteus, and gastrocnemius –the latter being the only which inserts distally to the calcaneus, all the other ones are attached to the tibial diaphysis. When women are sat with extended legs (which is the case for backstrap loom weaving except wool and in one case of embroidery), the quadriceps muscles (rectus femoris, vastus lateralis, vastus medialis, and vastus intermedius) guarantee the extension of the knee and all insert distally onto the patella.

#### Traditional embroidery.

**Fig 3:**
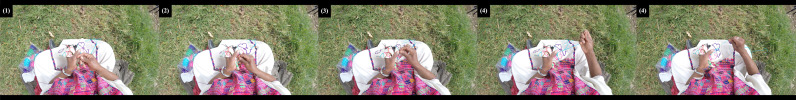
Motion sequence of traditional embroidery. (1) inserting the needle into the fabric; (2) pushing the needle with the thumb; (3) grabbing the needle with index and thumb; (4) pulling the thread two times. Photo credits: Alizé Lacoste Jeanson.

Traditional embroidery implies a series of four movements that the women repeat five to eleven times per minute (Fig 3):

- (1) inserting the needle into the fabric;
- (2) pushing the needle with the thumb;
- (3) grabbing the needle with index and thumb;
- (4) pulling the thread two times.

Right arm and hand make up all the dexterous movements required with the needle while left arm and hand hold the fabric in a constrained way that involves strength, especially at the elbow (stays flexed) and wrist level (stays neutral and supine). The left hand holds the same position in power grip, which implies the use of muscles extensor pollicis brevis, flexor digitorum profundus and flexor digitorum superficialis. The right hand grasps with precision, which involves the use of muscles abductor pollicis brevis, adductor pollicis, and flexor pollicis brevis—those insert respectively and distally at the level of the radial base of proximal phalanx of the thumb, at the ulnar proximal phalanx of thumb and at the palmar and ulnar base of proximal phalanx of the thumb. It should be noted that the thumb is the most active of all fingers in traditional embroidery; while all the other digits stay flexed (implying muscle flexor digitorum profundus, which insert distally at the base of distal phalanges of digits 2–5), the thumb goes from flexed to extended at the level of the interphalangeal joint (muscles extensor pollicis longus, extensor pollicis brevis, flexor pollicis longus, all attaching to the thumb distal phalanx). The left hand stays in a neutral position (nor pronate neither supine) while the right hand alternates between supination to pronation, which implies medial and lateral rotation and a slight ulnar deviation.

Movements of the right arm are: pronation-supination implying muscles inserting at the humeral epicondyles both lateral (muscles supinator, anconeus, extensor digitorum, extensor carpi, radialis brevis, extensor digiti minimi, and extensor carpi ulnaris) and medial (muscles flexor digitorum superficialis, pronator teres, flexor carpi radialis, flexor carpi ulnaris, flexor digitorum superficialis, and the palmaris longus); extension-flexion and horizontal abduction-adduction of the shoulder which imply the use of muscles biceps brachii (insertion distal on the radial tuberosity), supraspinatus and infraspinatus (both inserting on the humeral greater tubercle), pectoralis major, coracobrachialis, lastissimus dorsi, teres major and minor (all inserting on the humeral diaphysis).

#### Backstrap loom weaving.

**Fig 4:**
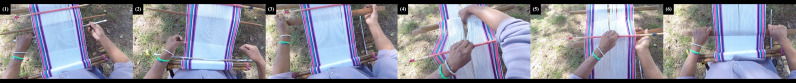
Motion sequence of backstrap loom weaving. (1) passing the shuttle with weft in between warp layers from left to right or right to left; (2) adjusting the thread at both lateral ends of the loom; (3) taking the batten out of the loom; (4) pulling the heddle rod up in the air and pulling the shed rod towards the front loom bar; (5) passing the batten through warp again; (6) pulling batten towards the body two to three times. Photo credits: Alizé Lacoste Jeanson.

As for backstrap loom weaving, action can be broken down in a series of six movements (Fig 4):

- (1) passing the shuttle with weft in between warp layers from left to right or right to left;
- (2) adjusting the thread at both lateral ends of the loom;
- (3) taking the batten out of the loom;
- (4) pulling the heddle rod up in the air and pulling the shed rod towards the front loom bar;
- (5) passing the batten through warp again;
- (6) pulling batten towards the body two to three times.

It should be noted that step (1) is ambidextrous: hands act as a relay between each other (the same movement is repeated for both sides as the thread needs to pass from one side to another and vice versa). The series of movements, with the exception of step (4), are repeated one to three times per minute, qualifying them for being repetitive movements. Step (4) comes only after having repeated one cycle of steps (1), (2), (3), (5) and (6). When pulling the heddle rod, the women who sits with buttocks on the ground (cotton weaving) need to exert a coxofemoral anteversion with the trunk going forward and buttocks toward the back—one woman just moves the pelvis with a slight help of her heels pushing the ground, while the other one actually help herself with a flexion of the leg at the level of the tibiofemoral joint pushing the ground with her heels, just like rowers do during the power phase of the stroke [24]. Women weaving with wool and sitting on their knees need to stand on their knees to realize the same movement, and to do so they raise their bodies with their toes pushing toward the ground thanks to a dorsiflexion posture. Weaving with wool also requires more strength and amplitude than weaving with cotton, as both loom and battens are much larger and heavier. A wider range of coxofemoral flexion is observed. Variation is also observed in the shuttle manipulation (step (1)): every woman seems to have adapted her own “style” which ranges from holding with three fingers, with power grip or in-between digits 2 and 3.

Kinematics implied in backstrap loom weaving with cotton or polyester involve a large range of motion of the upper body, which are summarized in Table 1. As compared to traditional embroidery, backstrap loom weaving involves extension of both elbows (only of the right one for embroidering) of elbow; more movements with ulnar deviation of the wrist; and wider flexion-extension of the wrist for both arms, which also require more strength because of the weight of the batten(s). Most of the hand grip is power, whereas for embroidery more precision grip (especially for the right hand) is observed.

**Table 1:** Description of upper limb’s joint, segment motion, relative muscular implication and bone distal insertion implied in both traditional embroidering and backstrap loom weaving.

Joint	Motion	Muscular implication and bone insertion
Shoulder	Horizontal abduction-adduction extension-flexion	Radial tuberosity: muscle biceps brachii Humerus greater tubercle: muscles supraspinatus and infraspinatus Humeral diaphysis: muscles pectoralis major, coracobrachialis, latissimus dorsi, teres major and minor
Elbow	Flexion-extension	Lateral humeral epicondyle: muscles supinator, anconeus, extensor digitorum, extensor carpi, radialis brevis, extensor digiti minimi, and extensor carpi ulnaris Medial humeral epicondyle: muscles flexor digitorum superficialis, pronator teres, flexor carpi radialis, flexor carpi ulnaris, flexor digitorum superficialis, and palmaris longus Radial tuberosity: muscle biceps brachii Ulnar coronoid process: muscle brachialis Ulnar olecranon process: muscle triceps brachii
	Pronation-supination (rotation)	Radial styloid process: brachioradialis Radial diaphysis: muscles pronator teres, pronator quadratus, supinator
Wrist	Palmar flexion-extension	Humeral medial epicondyle and metacarpal bases: muscles flexor carpi radialis, flexor carpi ulnaris, extensor carpi radialis longus, extensor carpi radialis brevis, and extensor carpi ulnaris Dorsal distal phalanx of thumb: muscle extensor pollicis longus Proximal phalanx of the index finger: muscle extensor indicis Proximal 5^th^ digit phalanx: muscle extensor digiti minimi Base of proximal and middle phalanges, digits 2–5: muscle extensor digitorum
	Ulnar deviation	Base of 5^th^ metacarpal bone: flexor carpi ulnaris and extensor carpi ulnaris
Hand and fingers	Power grip	Dorsal proximal phalanx of thumb: muscle extensor pollicis brevis Base of distal phalanx, digits 2–5: muscle flexor digitorum profundus Base of middle phalanx, digits 2–5: muscle flexor digitorum superficialis
	Precision grip	Radial base of proximal phalanx of the thumb: muscle abductor pollicis brevis Ulnar proximal phalanx of thumb: muscle adductor pollicis Palmar and ulnar base of proximal phalanx of the thumb: muscle flexor pollicis brevis

### Musculoskeletal injury’s risk

#### Standardized Nordic questionnaire of musculoskeletal symptoms.

None of the women have been hospitalized for problems related to embroidery or backstrap loom weaving. One woman fractured her wrist two years prior to the study (December 2023) but she does not report specific pain related to the accident. One woman works as a housekeeper and report high levels of pain in the entire body—for this reason she sits on a small chair for practicing embroidery but on the ground for weaving (as required by the craft). Interestingly, she does not report experiencing discomfort in different areas of the body compared to the other women. Another woman reports that a few years ago she had intense pain at the wrist level and left the weaving trade for a year, but backstrap loom weaving was not the cause for the pain. All the women went to see healers [*curanderos*] and “bone healers” [*hueseros*] to alleviate their pains, and have actually never been to a hospital for musculoskeletal symptoms.

The women we worked with all started embroidering between age 5–13. They practice traditional embroidery between one hour per day one day a week to six hours per day five times a week. The amount of time they practice is clearly reflected in the speed they make their stitches, which ranges from four to eleven times per minute—dexterity being higher when the trade is practiced more often and for longer times. 

As for backstrap loom weaving, the craft is started to be learnt around 13–14 years old. During adulthood, it is practiced between two and seven hours a day, three days a week during the dry season (half of the year). During the rainy season (from May to October in Chiapas), backstrap loom weaving is usually not practiced, as the main implements for working are an outdoor setting and the sunlight. The climatic and seasonal factor also applied for traditional embroidery as what is needed most is actually seeing well. It is usually not practiced indoors with artificial light.

All the women considered for the study about embroidery report symptoms at the level of the neck (cervical vertebrae) and of the right shoulder. The right arm is the one with which the thread is pulled. Most of the women (3 of 5) report pain at the dorsal and lumbar level of the back. All the women report discomfort in both hands (3 of 5 for the left hand, 4 of 5 for the right hand), mostly in the thumb and index of both hands, although some also report discomfort of third and fourth fingers.

As for backstrap loom weaving, more generalized symptoms in both arms from shoulder to fingers are reported by all the women. Interestingly, pain is reported in upper back (neck) and lower back (lumbar) for women who sit on their buttocks (cotton loom) whereas for women who sit on their knees (wool weaving), pain is reported only at the thoracic level—not in the lower back or neck.The latter group also report that the most intense pain is actually in the knees.

All the women report that pain or discomfort from the crafts never impeded them to practice such activities or other activities—they more so consider those symptoms as tiredness [*cansancio*], which disappear as soon as they stop embroidering or backstrap loom weaving. Nevertheless, constrained postures and repetitive movements entail a risk of producing injuries in body segments even though the person does not know that said injury is because of such exposure.

#### Repetitive movements.

A repetitive movement is defined by being a series of same or identical movements repeated various times per minute during a large time or when the same activity is practiced for more than 50% of the working day [7], which is the case for the women considered, more so for backstrap loom weaving, which amounts more hours a day that traditional embroidery. Repetitive movements are identified in the upper limbs (shoulders, elbow, wrists and fingers) for both crafts; and also, at the level of the knee for backstrap loom weaving.

#### Constrained postures.

Constrained postures are defined being either prolonged postures (static) and/or postures, which border limits of the mobility arch (dynamic) and have been evaluated following REBA method [9]. Constrained postures involve neck (>20° flexion) for traditional embroidery exclusively. For both crafts, constrained postures are observed at the level of pelvic girdle (trunk flexed 90° to 100°), lower limbs (knees >60° flexion in some cases), elbows (<60° flexion or > 100°), wrists (flexion>15° with deviation in some cases) and fingers. REBA’s results of postural load indicate that all postures present high (scores 8–10) to very high (scores 11–15) risk of injury, which indicate the need to take action immediately with the aim of reducing the risk level: traditional embroidery scores are between 8 and 12; backstrap loom weaving range 12–15.

### Skeletal changes

Differences between types of prehension (power grip and precision grip) used repetitively during occupational activities have been demonstrated to be reflected in hand entheses patterning where thumb muscles insert (Table 1 and [25]): precision grip implies the activation of a set of muscles used to position the thumb relatively to the fingers and palm while power grip involves mostly the use at thumb level of muscle extensor pollicis brevis and muscles flexor digitorum profundus and flexor digitorum superficialis, which participate in the biomechanics of flexion of all other fingers [25,26].

After years of practice and the onset of age-related bone degeneration,knee alterations are to be expected in individuals who weave with a backstrap loom using cotton (sat on the buttocks with extension and flexion of the knee joint in a horizontal plane). We can hypothesize that the patella would demonstrate either extension of its surfaces and/or osteophytes on its contours, or polishing of its articular surfaces. Patellar articular extension even more so applies to backstrap loom weaving with wool, as the posture implies loading the entire body on the knee. It could also be hypothesized a notched patella because of the extreme flexion of the knee when pulling the heddle rod up (step 4) and passing the batten through the loom (step 5).

Entheseal change at the medial epicondyle of the humerus, which corresponds to the origin of the common flexor, should also be expected. Changes at the ulnar coronoid process where inserts the ulnar collateral ligament could also be expected for backstrap loom weaving considering the repetitive flexion-extension of the elbow.

Repetitive movements in both upper and lower limbs—particularly at the shoulder, elbow, wrist, and knee—can lead to overuse injuries such as tendinitis, carpal tunnel syndrome, tenosynovitis, and bursitis. 

With regard to asymmetry of bilateral appendicular bones, it would be expected that more asymmetry would be developed at the humeral diaphysis for traditional embroidery, but a relative symmetry should be expected for backstrap loom weaving. No asymmetry at the level of the legs should be caused by textile craftsmanship.

Skeletal changes can manifest as musculoskeletal lesions such as osteoarthritis in certain individuals (e.g., [27,28]). In the case of traditional embroidery and backstrap loom weaving, it would be expected that osteoarthritis would develop more at the level of hand joints, wrist and back.

Backstrap loom weaving involves prolonged trunk and knee flexion, which increases spinal loading and contributes to degenerative conditions such as disc damage, joint dysfunction, and even vertebral fractures. Traditional embroidery also leads to frequent reports of neck and lower back pain due to sustained forward-flexed postures, which could cause similar skeletal lesions.

In backstrap loom weaving, additional strain on the pelvic girdle due to repetitive hip flexion and extension increases the risk of bursitis in that region.

## Discussion

This study examines the biomechanics of traditional embroidery and backstrap loom weaving in Chiapas, focusing on posture, movement patterns, and musculoskeletal risks. Both crafts involve prolonged static positions and repetitive motions, particularly affecting the upper limbs, back, and knees.

The Standardized Nordic Questionnaire revealed common discomfort in the neck, back, shoulders, hands, and knees, though artisans perceive symptoms as temporary fatigue rather than chronic injury. However, REBA scores indicate a high to very high risk of musculoskeletal strain, suggesting a need for ergonomic interventions.

The postural load is particularly increased by the knees (in some cases) and trunk (in all cases) being so flexed; the static aspect of the activities for various body parts (trunk, neck for both embroidery and weaving, and also legs for embroidery), the repetition of movements more than four times per minute in the case of traditional embroidery and large range changes in postures with or without an unstable base for backstrap loom weaving with wool as well as loading weight (the *machete* tool).

Those results are comparable to those obtained by Das and Singh (2022) on floor-sitting precision handicraft workers, who report prevalence of musculoskeletal symptoms in the neck, lower back and knee region [29]. It is also congruent with a study lead on traditional weaving in Chencha district, Gamo zone, Ethiopia in which—although the study does not describe the way weaving is actually practiced—most musculoskeletal disorders were found at the level of the shoulder, the low back and the neck, reinforcing the notion that prolonged sitting and years of practice exacerbate musculoskeletal strain [30]. A study among traditional weavers in Arunachal Pradesh, India, reported a high prevalence of musculoskeletal disorders, particularly in the lower back, with 79.2% experiencing trouble in the past 12 months. The study attributed this to prolonged work exposure and awkward postural demands, emphasizing the need for ergonomic interventions [31]. Similarly, research on traditional cloth weavers in Addis Ababa, Ethiopia, found a high prevalence of low back pain associated with factors such as working more than 8 hours per day, frequent bending, uncomfortable postures, and tasks that exert pressure on the back [32].

Studies on rowers, who engage in repetitive lumbar flexion and extension, show a high prevalence of low back pain due to excessive spine loading—paralleling the strain observed in backstrap loom weavers [24,33]. Low back pain because of excessive lumbar spine loading (induced by both constrained position of the trunk flexed 90° to 100° and repetitive flexions of the knees) is an important risk factor for backstrap loom weaving. In rowing, the repetition of lumbar flexion and extension, especially in the absence of adequate pelvic movement, has been closely associated with a high prevalence of low back pain. It has been identified a common attribute in rowers with training sessions longer than 30 minutes—a threshold commonly surpassed in traditional weaving practice [33].

For traditional embroidery, low back pain and neck pain due to constrained position of both trunk and neck (>20° flexion) is actually reported through the Standardized Nordic questionnaires.

Low back pain could be the result of annular tears, disc degeneration, facet disease and sacroiliac joint dysfunction [24]. Herniation and spondylolysis (fracture of pars interarticularis) at the lumbar vertebrae could be expected as those conditions are observed with high frequency in athletes practicing rowing as compared to the general population—the latter at a frequency of 17% in rowers and 5% in the general population—and associated with hyperextension of the arms in rowers, similarly observed in backstrap loom weaving [34,35].

Since both crafts considered in the current paper imply a high ergonomic risk factor, they might lead to injuries and as such it is relevant to assess skeletal changes providing the frequent repetition of motions executed and the constrained postures of the body.

The repetitive nature of these tasks at the upper and lower limbs implying shoulder, elbow, wrist and knee increases the likelihood of conditions such as: tendinitis (tendon’s inflammation, which is common at the shoulder, elbow, and knee); carpal tunnel syndrome (compression at the wrist’s level of the median nerve, which itself goes from forearm); tenosynovitis of the wrist, elbow and of the knee (inflammation of the sheath that surrounds tendons); bursitis of shoulder and knee (inflammation of bursae, which contains the synovial fluid and acts as a shock absorber between bone, muscles and tendons). Bursitis would also be expected at the level of the pelvic girdle in the case of backstrap loom weaving, as one of the movements implies its repetitive flexion and extension (movement (4): pulling the heddle rod up in the air and pulling the shed rod towards the front loom bar).

Expected skeletal changes include entheseal changes at muscle attachment sites, particularly in the hands and arms, with potential long-term effects (lesions).

Skeletal changes seen in repetitive manual laborers, such as joiners, shoemakers, and tailors, suggest that power and precision grip patterns leave distinct entheseal markers [25]. Similar patterns are expected in the Chiapas artisans, where embroidery likely results in more pronounced changes in the dominant hand, while weaving should lead to more symmetrical modifications.

Embroidery relies heavily on precision grip, while weaving incorporates both precision and power grips, with wool weaving requiring greater force and flexibility. It should be expected then that entheseal surface at the dorsal proximal phalanx of the thumb, dorsal entheseal surface situated next to the head of first metacarpal and proximal base of digit 2 proximal phalanx should be more developed relative to the other set of insertion sites of muscles implied in precision grasping. As for power grip, it would be reflected in relatively more developed entheseal surfaces of proximal phalanges of thumb and digit 5, as well as base of the 5^th^ metacarpal and base of distal phalanx.

The knees are very engaged in backstrap loom weaving with cotton, in a way that compares to the movements of the rowers, in which syndromes are observed at the patellofemoral joint, and can lead to dislocation [33]. Changes of the patella is thus expected for this activity. Expanding upon the comparison between traditional textile crafts and rowing, both activities involve repetitive motions and sustained postures that can lead to specific musculoskeletal issues. In rowing, the constant flexion and extension of the knee under load can lead to conditions like chondromalacia patella and iliotibial band friction syndrome, which could also be expected for backstrap loom weaving with cotton. Additionally, the necessity to feather the oar results in extensor tenosynovitis [24].

Furthermore, lower limb modifications from backstrap loom weaving with wool (on the knees) could resemble patterns found in individuals engaged in kneeling-intensive tasks; notched patellas have actually been found in the archaeological record in women grinding on large grinding stones and sitting in the same posture as backstrap loom weaving with wool [36].

As for the other joints involved in the movements of textile crafts and reported musculoskeletal symptoms, it can be hypothesized skeletal changes at the level of the vertebras and the humerus.

Entheseal changes at the medial epicondyle of the humerus may also manifest in weavers because of the repetitive strain of shuttle manipulation. It is commonly referred to as golfer’s or tennis players’ elbow [37] and has also been found in a cello player [38] and medieval crafts [39]. Affliction (neuropathy) of the ulnar collateral ligament (insertion at the ulnar coronoid process) has also been observed in rowers [24], which could be compared to arms motion in backstrap loom weaving.

For both activities, osteoarthritis, if occurring, is likely to develop primarily in the hand joints, wrists, and back. However, it should be noted that osteoarthritis, and osteoarticular lesions in general, is not ultimately a risk corresponding to repetition of specific sets of movements; it is actually a maladaptation of the skeleton to both physical and biological pressures [40–42]. Studies of Wallace et al., both experimental and set in past and contemporary populations, demonstrate that prevalence of knee osteoarthritis actually correlates with increase in rich food intake during the lifecourse [40–42].

In the case of the current investigation, it should be added that Highland Chiapas, because of its mountainy elevation, experiences cold weather at nights and during the winter as well as half year of intense rains (exemplified by 1380.4 mm of water per year in average for the years 1991–2020 in the city of San Juan Chamula according to the Mexican Water National Commission). Both attributes (cold and humidity) are known to increment the risk of osteoarthritis, or at least the felt pain of this musculoskeletal symptom [43,44].

These results align with previous research on occupational, sports and craft-related biomechanical strain. Those studies recommended ergonomically oriented weaving workstations to alleviate these issues [32]. There is, however, a fundamental difference between those studies and ours: the crafts we described are practiced for only a few hours a day, exclusively in natural light, and during certain times of the year. Despite its physical demands, the work remains within manageable limits, as women work autonomously and are free to stop as needed.

## Conclusion

The case-study conducted in the Highlands of Chiapas, Mexico, provides a comprehensive understanding of the musculoskeletal risks associated with traditional backstrap loom weaving and embroidery. Through detailed kinematic and biomechanical analysis, it was estimated that the musculoskeletal injury risk level for women engaged in these crafts is high to very high. The primary risk factors include the frequent repetition of the same movements, the adoption of constrained postures (particularly in the trunk, neck, and legs), and the positioning of the legs while sitting on the floor during both traditional weaving and embroidery. The observed leg positions—whether fully extended with a flexed trunk or cross-legged/sitting on knees—contribute to prolonged strain on the lumbar spine, hips, and knees.

The Standardized Nordic questionnaires confirmed the diagnostic, with the majority of women reporting musculoskeletal symptoms in both arms, back and neck. Skeletal changes from backstrap loom weaving and traditional embroidery based on detailed analysis of muscles implied in all motions observed, and comparisons with sport and occupational medicine data indicate that entheseal changes would be expected at the humeral medial epicondyle and at the ulnar coronoid process. In the particular case of backstrap loom weaving with wool (sitting on the knees), entheseal change at the patella as well as extension of the patella femoral joint (for both styles of weaving) could be expected. As most motions require power grip of both hands, entheseal surfaces of the fingers corresponding to flexion of all fingers (power grip) would be observed symmetrically for both hands for backstrap loom weaving but unilaterally for traditional embroidery (the left hand in the case of right-hand people).

Various pathologies could result from such ergonomic diagnostic: tendinitis carpal tunnel syndrome, tenosynovitis, bursitis for the upper limb and pelvic girdle; as well as annular tears, disc degeneration, facet disease, sacroiliac joint dysfunction, herniation and spondylolysis for the spine. These findings underline the significant risk of long-term musculoskeletal pathologies resulting from the ergonomically demanding nature of these traditional crafts.

In order to further assess the actual skeletal changes and lesions caused by textile craftsmanship, the current investigation aims to study bones of deceased people who lived in the same geographical area during the Classic Maya period (AD 200–900), and who were buried with tools suggesting practice of those crafts.

Despite the ergonomic concerning findings, the value of this study extends beyond documenting health risks. It offers critical insights into how the practice of traditional crafts—which are both economically significant and culturally important—can be sustained in a way that minimizes musculoskeletal injury risk. Moving forward, it is imperative to refine the ergonomic analysis further, and to develop targeted preventive strategies. These strategies should be based on the specific movements and postures identified in this study and may include:

1. Improved workstation design: ergonomically adapted looms and seating arrangements (e.g., adjustable-height stools, knee cushions) that promote neutral body positioning and reduce strain on the spine, upper limbs, and legs.
2. Postural education: implementing educational programs for women artisans on the importance of posture correction, and stretching routines to alleviate strain.
3. Health surveillance and early intervention: establishing programs for early detection and treatment of musculoskeletal disorders through routine health assessments and ergonomic counseling.

However, it is important to emphasize that while the ergonomic risks are high, the traditional practices of backstrap loom weaving and embroidery in the Highlands of Chiapas are ancient, culturally significant, and are carried out with a high degree of individual autonomy and self-management, as opposed to workers of the global textile industry. As noted in the results section based on the Standardized Nordic Questionnaires, the women involved in these practices do not engage in them for extended periods. Most women practice these crafts for only a few hours a day, only during daylight, and at specific moments of the year. The activities, though physically demanding, are not performed to the point of physical breakdown—the participants of our study are never forced to continue the work. Rather, these crafts hold significant cultural value, and while the physical strain is evident, the balance between work and rest, along with the voluntary nature of these activities, is an important factor in their sustainability and cultural significance.

The findings from this study not only highlight the importance of addressing the ergonomics of traditional labor but also underscore the need for culturally sensitive interventions that support artisans’ health while preserving their traditional skills. Through preventive measures, ergonomic interventions, and continued research, it is possible to reduce the musculoskeletal risks associated with these crafts, ensuring that textile craftsmanship remains both a sustainable practice and a healthy livelihood for future generations.

Overall, these findings highlight the need for further investigation into the long-term skeletal effects of traditional textile craftsmanship. Future research should incorporate skeletal analyses, ergonomic interventions, and cross-population comparisons to better understand the implications of these crafts on musculoskeletal health while ensuring the sustainability of these cultural practices.

## Supporting information

S1 Checklist Inclusivity in global research. (PDF)
