# Supplementary material for: Assessing musculoskeletal injury risk and skeletal changes from backstrap loom weaving and traditional embroidery in Chiapas, Mexico
Source: PLOS Glob Public Health. 2025 Apr 29;5(4):e0004574. doi: 10.1371/journal.pgph.0004574 (PMC12040135; doi:10.1371/journal.pgph.0004574)
Supplement: S1 Checklist — (PDF) [file pgph.0004574.s001.pdf]

Inclusivity in global research

PLOS' policy on inclusivity in global research aims to improve transparency in the reporting of research performed outside of researchers' own country or community and ensures that PLOS publications reporting global research adhere to high standards for research ethics and authorship. Authors of relevant research articles may be asked to complete the questionnaire below, which outlines ethical, cultural, and scientific considerations specific to inclusivity in global research. This questionnaire may be requested when researchers have travelled to a different country to conduct research, if research uses samples collected in another country, research with Indigenous populations or their lands, or if research is on cultural artefacts. Researchers travelling to another country solely to use laboratory equipment will not normally be required to complete the questionnaire. However, the questionnaire can be requested at the journal's discretion for any submission – if you have been requested to complete this questionnaire by the PLOS journal you submitted to, please do so.

Please complete the questionnaire below and include this as a Supporting Information file with your manuscript. Note that if your paper is accepted for publication, this checklist will be published with your article in the supporting information files. Please ensure that you reference the checklist in the main body of your manuscript. We suggest adding a subsection 'Inclusivity in global research' to your Methods section and adding the following sentence: "Additional information regarding the ethical, cultural, and scientific considerations specific to inclusivity in global research is included in the Supporting Information (SX Checklist)"

The questions have been designed to be applicable to a wide range of study types, and there are subsections for both human subjects research and non-human subjects research. If any of the questions are not relevant to your research please mark them as "N/A" as appropriate.

Ethical considerations, permits and authorship

This section is applicable to all research types.

Provide details as to who granted permissions and/or consent for the study to take place in the Methods section of your manuscript. This should include the names of **all** ethics boards, governmental organizations, community leaders or other bodies that provided approval for the study. If individuals provided approval refer to these people by their role or title but do not list their name(s).

Reported on page number: 6

If there were any deviations from the study protocol after approval was obtained please provide details of these changes in the Methods section of your manuscript.

Reported on page number: does not apply

Did this study involve local collaborators that are residents of the country where the research was conducted or members of the community studied? If you do not have any authors from said communities, please provide an explanation for this below.

Yes, I am myself (Alizé Lacoste Jeanson) resident of Mexico and have been living in Chiapas (San Cristobal de las Casas) for more than 6 years. My two co-authors are Mexican and resident of Mexic City. All of the women who participated in the study are residents of San Cristobal de las Casas and San Juan Chamula and are my actual neighbours.

Everyone listed as an author should meet PLOS' criteria for authorship and all individuals who meet these criteria should be included in the author byline, rather than the acknowledgements. For further information please see the journal's Authorship Policy.

Human subjects research (e.g. health research, medical research, cross-cultural psychology)

Did you obtain written informed consent from a representative of the local community or region before the research took place? How did you establish who speaks for the community? Details of written informed consent obtained from study participants should be reported separately in the Methods section of your manuscript.

I obtained informed consent from all of the women who participated in the study, who are all autonomous beings, residents of the cities of San Cristobal de las Casas and of San Juan Chamula and most of them founding members of an organization in which they organize themselves, practice backstrap loom weaving and embroidery and cultivate a garden and/or a cultural center whci aims to re-dignificate the history of native people. There is no need of representative of the local community (which are actual cities) to speak for the women who participated in the study, those are actually deficient, corrupted and macho, hence the need of auto-organize as a grassroot level.

How did members of the local community provide input on the aims of the research investigation, its methodology, and its anticipated outcome(s)?

I actually designed this research with members of the local community, fact that we reported with Rocío del López de la Cruz in two speeches in scientific meetings and one book's chapter that's about to be published (attached). This piece is part of a bigger community investigation on body modification and textile production, led by La Tierra del Maíz and partly supported by the grant I obtained from the Mexican National Council of Sciences. We saw each other and talked on various occasions before I started. We are now working together in organizing meetings which aim to share knowledge about textile production and body modifications (see posters attached) both from an academic viewpoint and a community viewpoint.

When engaging with the local community, how did you ensure that the informed consent documents and other materials could be understood by local stakeholders?

We speak the same language: Spanish. Two of the women only spoke Tsostil and their daughter-in-law and daughter, respectively, translated the document as well as the questions of the Standardised Nordic Questionnaire. All of the process has been filmed.

Will the findings of the research be made available in an understandable format to stakeholders in the community where the study was conducted (e.g. via a presentation, summary report, copies of publications, etc.)? Please provide details of how this will be achieved.

As said, we are organizing events to share the results of our community investigation, which is both on textile production and body modification. In those events, we present speeches, animate workshops and most women who have participated in this part of the investigation are either part of the organization of said events or assistents. I also shared with them the paper that I submitted to PLOS before submitting it and they helped me with recent historical background (beginning of introduction section) and name of the tools (Fig 2). They all have in their possession the paper translated to Spanish with DeepL. For the two women who only speak Tsotsil (which I'm actually learning), I see them very often since I'm part of their network through which we dstribute their textiles and we have spoke about the results through their daughter-in-law and daughter.

Non-human subjects research using specimens/ animals collected as part of the study, or those housed in archival collections. Examples include archaeology, paleontology, botany and zoology.

Did the permission you obtained from a local authority to perform the study include an agreement on access to outputs and benefit sharing? This may include procedures to enable fair distribution of the benefits and resources arising from the research performed. Please include any details of Prior Informed Consent and Benefit Sharing Agreements obtained. These may be required by field-specific regulations, for example the Convention on Biological Diversity (CBD) and the associated Nagoya Protocol.

Does not apply.

If the material used in your study was imported, please A) provide the year it was imported and B) indicate whether permits were obtained to import/export the materials used, C) provide details of any permits obtained. If this information is not available, please indicate this.

Does not apply.

If you used archival specimens, please state how the material used in your study was acquired by the institute it is held in and provide details of any permits obtained for the original excavations/ sample collection. If this information is not available, please indicate this.

Does not apply.

How was the potential cultural significance of the materials collected in your study to local communities considered in your research design? Were Indigenous peoples and/or local researchers and institutions involved with archaeological excavations / collection of specimens? If so, please provide a description of their involvement.

Does not apply.

If your manuscript includes photographs of human remains please indicate whether authors obtained permission from descendants or affiliated cultural communities to do so.

Does not apply.

UNIVERSIDAD NACIONAL AUTÓNOMA DE MÉXICO
INSTITUTO DE INVESTIGACIONES HISTÓRICAS

MUJERES

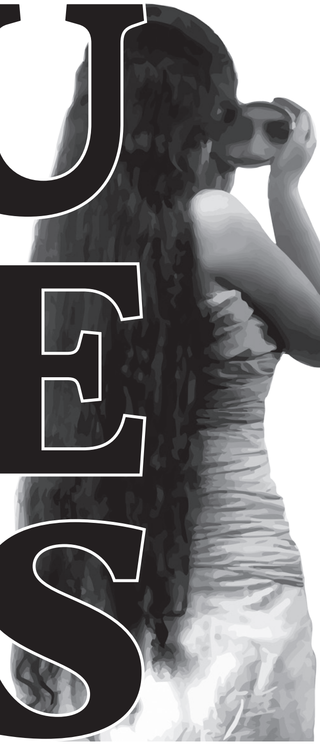

REGISTRO Y MEMORIA

PRIMERA JORNADA ACADÉMICA

22 y 23
de noviembre de 2023
Salón de Actos
Evento presencial

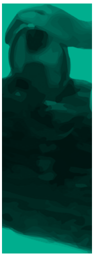

Estas jornadas reúnen a un grupo de académicos para discutir, desde sus propias áreas y temas de estudio, los registros y la memoria histórica que poseemos de las mujeres en diversos periodos y regiones, con la finalidad de abrir un debate relacionado con el uso y aprovechamiento de las fuentes que tenemos a nuestra disposición en la reconstrucción y devenir histórico de las mujeres y sus roles en la sociedad.

MIÉRCOLES 22 DE NOVIEMBRE

10:00

INAUGURACIÓN

10:15

CONFERENCIA MAGISTRAL

¿Dónde estaban las mujeres en el mundo prehispánico? Pintura mural teotihuacana y otros recorridos

María Teresa Uriarte UNAM, Instituto de Investigaciones Estéticas

11:00-12:30

MESA 1

El tejido de la historia: de los huesos al cosmos

Alizé Lacoste Jeanson y Claudia del Rocío López de la Cruz

UNAM, Instituto de Investigaciones Antropológicas

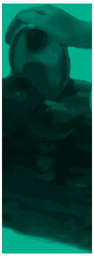

Las mujeres y las relaciones de género en la América colonial: fuentes, métodos y desafíos

Diana Roselly Pérez Gerardo UNAM, Instituto de Investigaciones Históricas

Señoras, sabias y guerreras: mujeres, autoridad y moral en el islam temprano y clásico

Felipe Cobos Alfaro UNAM, Posgrado en Historia

Mujeres historiadoras del siglo xx

Evelia Trejo UNAM, Instituto de Investigaciones Históricas

12:30

RECESO

12:50-14:30

MESA 2

Las mujeres en la Edad Media: una aproximación teórico-metodológica

Martín Ríos Saloma UNAM, Instituto de Investigaciones Históricas

Entre tinta y papel. Las trabajadoras de las imprentas en el siglo xix

Rosalba Cruz UNAM, Instituto de Investigaciones Históricas

Mujeres y migración en la segunda mitad del siglo xx

Diana Irina Córdoba UNAM, Instituto de Investigaciones Históricas

Jueves
23 de noviembre

10:00-11:45

MESA 3

Fundadoras y legitimadoras. El caso de Ilancueitl y Atotoztli

Clementina Battcock Instituto Nacional de Antropología e Historia

Las mujeres frente al Provisorato de Indios y Chinos del Arzobispado de México, siglo XVIII

Gerardo Lara Cisneros UNAM, Instituto de Investigaciones Históricas

Mujeres en la Nueva España

Estela Roselló Soberón UNAM, Instituto de Investigaciones Históricas

Mujeres empoderadas: formas de la asistencia social en los siglos XIX-XX

María Dolores Lorenzo UNAM, Instituto de Investigaciones Históricas

11:45

RECESO

12:00-13:00

MESA 4

Las mujeres en el mundo antiguo: ideales de representación

María Elena Vega UNAM, Instituto de Investigaciones Históricas

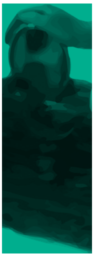

Marion Bryce, una británica de viaje por el México de Díaz

José Enrique Covarrubias UNAM, Instituto de Investigaciones Históricas
Itzel Toledo Lateinamerika-Institut, FreieUniversität Berlin

Derechos civiles y la mujer mexicana-americana en el tránsito del siglo XIX al XX

Silvestre Villegas UNAM, Instituto de Investigaciones Históricas

13:00

CONFERENCIA MAGISTRAL

Mujeres y cambio político

Ana Carolina Ibarra UNAM, Instituto de Investigaciones Históricas

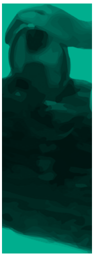

INSTITUTO DE INVESTIGACIONES HISTÓRICAS

Dirección General de Asuntos del Personal Académico

IGUALDAD DE GÉNERO UNAM

Se otorgará constancia de asistencia previa inscripción:
mujeres.registro.y.memoria@gmail.com

Circuito Mtro. Mario de la Cueva s/n, Zona Cultural
Ciudad Universitaria, Coyoacán, Ciudad de México

historicas.unam.mx

Tejer la historia: entre huesos y textiles

Alizé Lacoste Jeanson[1]

Claudia del Rocío López de la Cruz[2]

Los huesos y los textiles son tejidos, de fibras minerales para el primero, de fibras vegetales para el segundo. Esos hilos que nos estructuran y nos visten cuentan quienes somos, cada uno a su manera. Si bien los huesos registran la historia personal, han conformado la arquitectura biológica durante la vida mediante su adaptación a necesidades biomecánicas y biológicas, los textiles también registran la historia de los pueblos que les tejen a través de la selección de materiales, de los colores, de los símbolos. Este texto tejido a cuatro manos intenta ser la demostración de que la historia no solamente se lee en los textos escritos –la historia se puede leer desde los hilos, los más íntimos. La lectura de la historia a través del esqueleto y de las prendas permite dejar a un lado la narrativa hegemónica de quienes tienen el control de la escritura y de su difusión. Podemos contar a partir de nuestros huesos y de nuestras segundas pieles quienes somos y así, darle dignidad a la historia de nuestros pueblos y, entre ellos, de las mujeres y de los hombres que entretejen sus historias personales desde el pasado hasta hoy.

---

[1] Alizé Lacoste Jeanson estudió Filosofía, Etnología y luego Bio-Antropología en Francia. Se especializó en la Biomecánica del Esqueleto después de haber desarrollado un interés en las problemáticas de identificación humana a partir de los huesos. Es investigadora y responsable del proyecto de investigación "Tejido Óseo Maya" en el Instituto de Investigaciones Antropológicas de la UNAM.

[2] Claudia del Rocío López de la Cruz estudió Literatura Hispano-Americana con especialidad en Lenguas Originarias en Chiapas. Formó el grupo de estudio "Muk' Ta Luch" [El Gran Bordado] cuyo propósito es lucir las historias contadas por los textiles a través notablemente de la iconografía. Es fundadora del colectivo cultural "La tierra del maíz" en San Cristóbal de las Casas, Chiapas. Colabora con organizaciones sociales y, además, trabaja en la recaudación de la tradición oral.

TEJIDO ÓSEO

El hueso es un tejido conjuntivo compuesto de fibras de colágeno, de glicoproteína y de células (**Figura 1**).

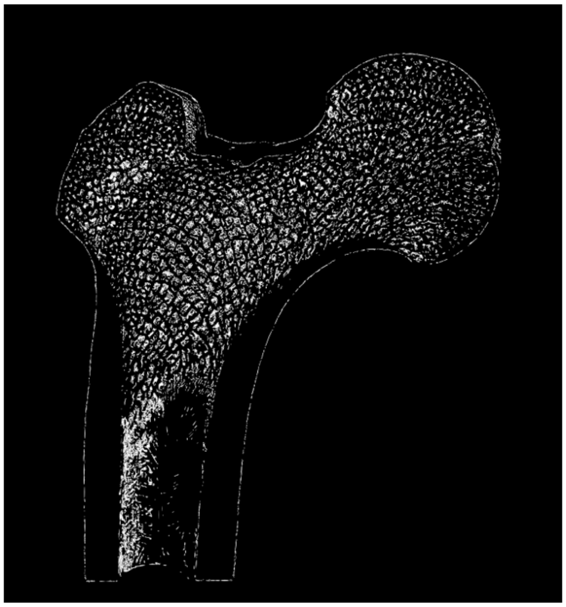

Figura 1: sección longitudinal de la cabeza femoral ©Alizé LJ, digitalizado a partir de Poirier, 1896[3]

El tejido óseo tiene la particularidad de mineralizarse; su matriz se carga de minerales que se amarran a las fibras de colágeno. El hueso es un tejido dinámico y mecano-sensible que busca la homeostasis –eso lo hace modificarse a lo largo de la vida para adaptarse a las necesidades vinculadas con el medio ambiente o las presiones biológicas. La adaptación funcional del esqueleto le permite mantener su integridad estructural y mecánica a través de procesos de remodelación que consisten en adición, resorción o sustitución de material óseo[4,5].

---

[3] Poirier, P. (1896). *Traité d'anatomie humaine. Tome Premier : Embryologie, Ostéologie, Arthrologie*. Masson.

[4] Frost, H. M. (1988). Vital biomechanics: Proposed general concepts for skeletal adaptations to mechanical usage. *Calcified Tissue International*, *42*(3), 145–156. https://doi.org/10.1007/BF02556327

[5] Ruff, C. B., Holt, B. M., Sládek, V., Berner, M., Murphy, W. A., Jr., zur Nedden, D., Seidler, H., & Recheis, W. (2006). Body size, body proportions, and mobility in the Tyrolean "Iceman". *Journal of Human Evolution*, *51*(1), 91–101. https://doi.org/10.1016/j.jhevol.2006.02.001

Registro de la información genética

Mediante esa función adaptativa del tejido óseo, la antropología biológica trata de identificar elementos biográficos a partir de los huesos, como el sexo biológico.

La pelvis es el único conjunto óseo del cuerpo humano vinculado con una función discriminante entre los sexos: la posibilidad exclusivamente femenina de parir. Por eso, los huesos coxales que constituyen parte de la pelvis (con el sacro) tienen dimensiones relativas y formas distintas entre los hombres y las mujeres (**Figura 2**).

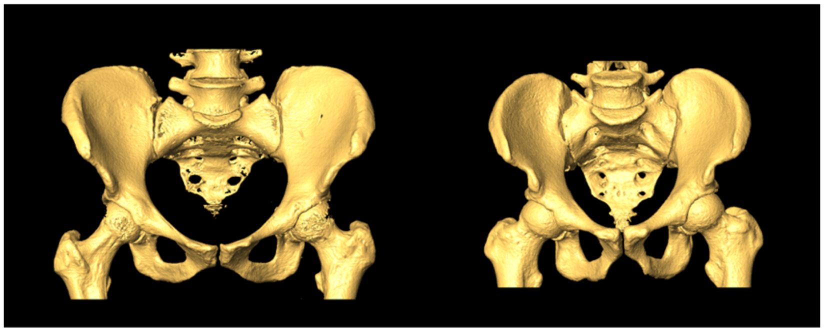

Figura 2: pelvis femenino (izquierda) y masculino (derecha) ©Alizé LJ

Es así que los huesos registran parte de la información genética que se manifiesta a nivel físico.

Registro del crecimiento y del desarrollo

Los organismos biológicos se modifican acorde a procesos ontogénicos de crecimiento y de maduración. Inician al momento de la primera división celular que dará lugar a un feto en el caso de los organismos multicelulares. Por disminución de la renovación celular, suceden procesos de senescencia para los que sobreviven hasta la edad adulta. Finalmente, la falta de oxigenación conduce a la muerte biológica.

El esqueleto está sometido a los procesos biológicos; se modifica también a lo largo del crecimiento, de la maduración y de la senescencia. Al momento de nacer, el cuerpo de los animales vertebrados está compuesto de más huesos que a la edad adulta: eso se debe a que los huesos necesitan tiempo de vida post-natal para terminar desarrollándose, crecer y terminan fusionándose. La bóveda craneal de los neonatos está compuesta de varios elementos óseos separados (**Figura**

3); las partes cartilaginosas se van osificando hasta dos años o un poco más de vida post-natal[6]. La última parte en fusionarse es la sutura esfeno-occipital que se junta (sincondrosis) hasta la adolescencia. Los huesos largos, como la tibia, también están compuestos de varias partes al nacer: varias epífisis (extremidades articulares) y una diáfisis (parte larga del hueso, en forma de viga), que se van juntando durante la infancia.

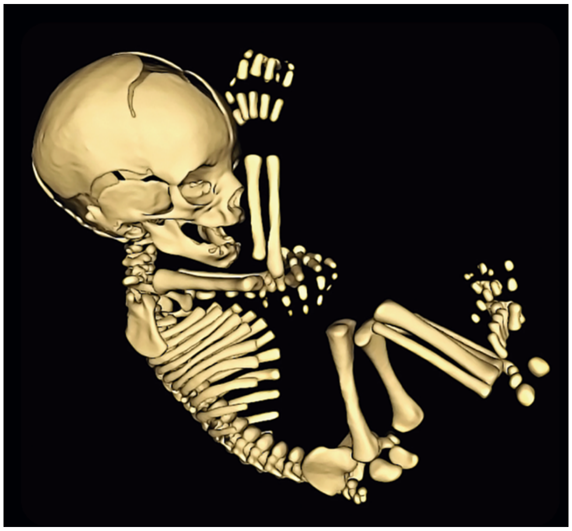

Figura 3: feto entre 37 y 39 semanas ©Paloma L. Gutiérrez González (proyecto PAPIIT IN402720 dirigido por Abigail Meza Peñaloza)

Es así que los huesos registran la edad de los individuos que estructuran.

Registro de la relación con el entorno

Los huesos largos como el fémur tienen una morfología adecuada a patrones de locomoción específicos: su morfología interna y la forma general de sus articulaciones están adaptadas a funciones locomotoras específicas especificas. Los fémures registran formas diferentes de moverse entre los grandes simios: los chimpancés caminan sobre sus nudillos; los orangutanes se desplazan mediante la braquiación-suspensión; y los humanos que practican la bipedestación de forma exclusiva (**Figura 4**).

[6] Scheuer, L., & Black, S. (2000). *Developmental Juvenile Osteology*. Academic Press.

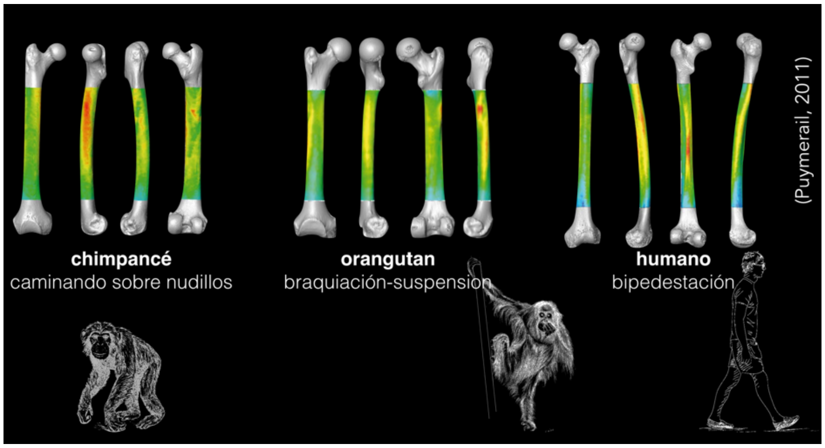

Figura 4: firma del modo de locomoción en los fémures de varios primates ©Alizé LJ a partir de Puymerail, 2011[7]

Es así que los huesos registran datos sobre la relación de los vertebrados con su entorno (ecología).

Registro de la actividad física

Los huesos sometidos a estrés extremos, pueden llegar a romperse. Pero las actividades repetitivas y que conllevan una cierta intensidad también llegan a modificar al esqueleto de una forma más sutil. Los lugares esqueléticos donde se amarran los músculos y los ligamentos se llaman entesis. Estas entesis llegan a modificarse a lo largo de la vida, parcialmente, en función de actividades físicas que implican cierta intensidad o repetición. Por ejemplo, en la Europa prehistórica, se ha demostrado que las mujeres estaban, por lo general, más involucradas en actividades manuales

[7] Puymerail, L. (2011). *Caractérisation de l'endostructure et des propriétés biomécaniques de la diaphyse fémorale: La signature de la bipédie et la reconstruction des paléo-répertoires posturaux et locomoteurs des hominines* (R. Macchiarelli & F. Marchal). Muséum national d'Histoire naturelle.

bilaterales (con los dos brazos) que los hombres –ellos presentan patrones de cambios entésicos al nivel del epicóndilo medial del húmero más de un lado que del otro[8] (**Figura 5**).

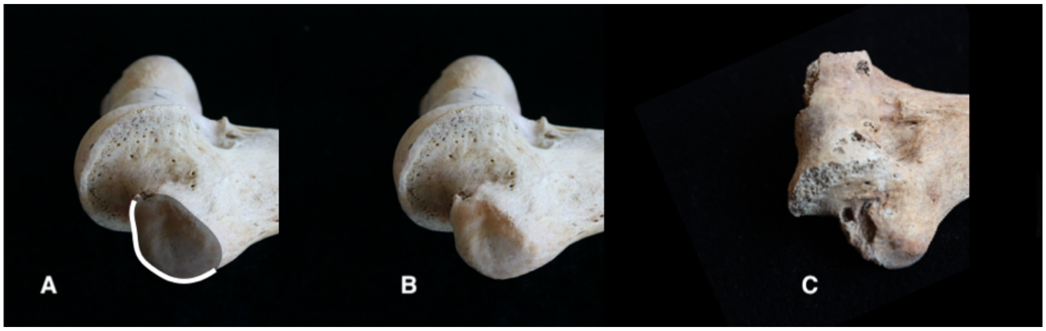

Figura 5: epicóndilo medial del húmero; (A) ubicación, (B) no modificado, (C) modificado ©Alizé LJ

Es así que los huesos registran parte de las actividades practicadas a lo largo de la vida.

•••

En teoría, al nivel bio-antropológico, se pueden entonces identificar las huellas dejadas por las actividades individuales del tejido, bordado e hilado en los esqueletos. Identificar estas prácticas en los huesos de personas del pasado nos permite remitir a elementos tanto de su vida cotidiana, de la división sexual de la labor como de su cosmología.

Si bien la práctica del tejido, del bordado y del hilado se registra a través del tejido óseo, también se registra en sus producciones. El tejido textil registra también la historia colectiva e individual y funciona como textos que se pueden alcanzar a descifrar. Al nivel cultural, esa lectura permite conectar los hilos de la memoria a través del reconocimiento de los procesos y de las personas que conciben los textiles a fin de dignificar la historia tanto personal como la de los pueblos.

[8] Villotte, S., & Knüsel, C. J. (2014). "I sing of arms and of a man…": Medial epicondylosis and the sexual division of labour in prehistoric Europe. *Journal of Archaeological Science*, *43*, 168–174. https://doi.org/10.1016/j.jas.2013.12.009

TEJIDO TEXTIL

Registro de la cosmovisión

Para los pueblos originarios de Chiapas, el textil forma parte de un registro cosmológico ancestral. Entre las prendas que usan hombres y mujeres se narran historias míticas de la creación.

En la lengua originaria *bast´i´kop* (tsotsil) existe un concepto conocido como *muk ʼta luch* que se traduce al español como "el gran bordado"; relacionado con la creación del universo, la naturaleza y el ser humano. El universo está compuesto por hilos que se van tejiendo para sostener al cosmos y a los seres que habitan el mundo terrenal.

Un huipil tradicional está diseñado de manera cuadrada, símbolo maya que hace alusión a los cuatro rumbos, los cuatro pilares que sostienen el mundo, que sostienen al ser humano. Su forma cuadrada no es casualidad, en su centro se forma el cuello del cual se emerge, , lo cual está relacionado con la metáfora del nacimiento y/o del renacimiento (**Figura 6**).

> "Cuando una mujer maya se pone su huipil emerge a través del cuello, simbólicamente, en el eje del mundo. Los dibujos del universo irradian de su cabeza, extendiéndose sobre las mangas y el corpiño de la prenda para formar una cruz abierta con la mujer en medio. Aquí se encuentran lo sobrenatural y lo ordinario. Aquí, en el mismo centro de un mundo tejido a partir de sueños y mitos, ella permanece entre el cielo y el inframundo."[9]

[9] Morris, Walter S Jr. *Diseños e iconografía: Chiapas*. México, D.F.; Tuxtla Gutiérrez, CHIS.: Dirección General de Culturas Populares; Consejo Estatal para la Cultura y las Artes de Chiapas, 2009:19.

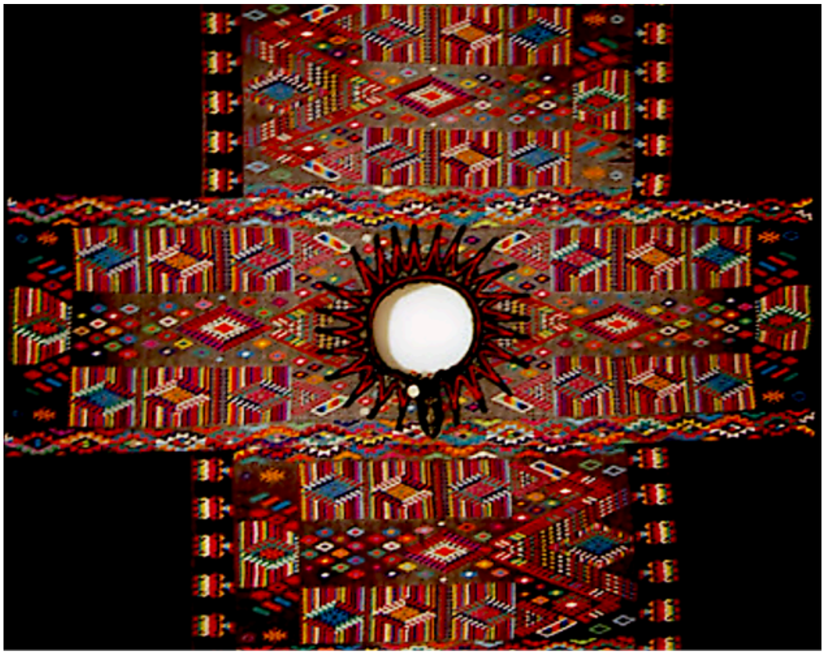

Figura 6: huipil guatemalteco

Registro de lo sagrado

Para los antiguos mayas, el textil no estaba separado de los conceptos sagrados. La diosa *Ixchel* o *Chul´metik* en *bats´i´kop* aparece repetidamente entre las diferentes narrativas antiguas y contemporáneas, ella, es la que revela las formas y colores que se deben bordar y/o tejer (**Figura 7**). Mujeres guardianas de historias antiguas y sagradas, siguen rezando y poniendo velas a *Chul´metik*/Diosa de la Luna y de la Tierra para que les regale el don de bordar y tejer.

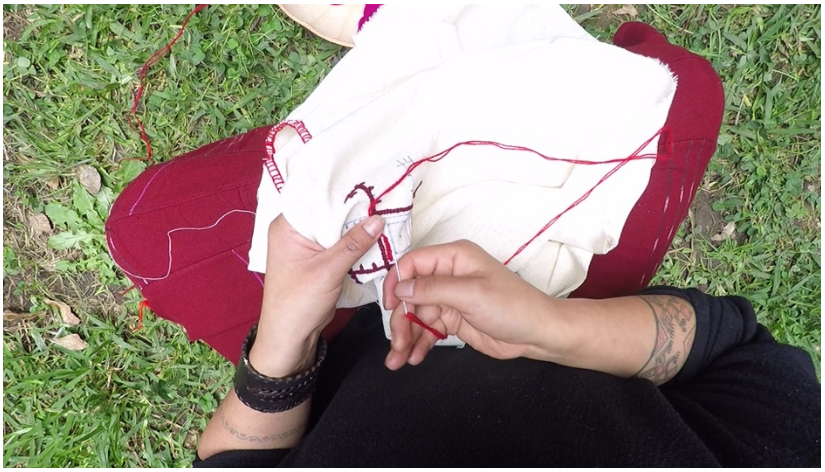

Figura 7: proceso de bordado de una Chul´metik en los Altos de Chiapas, 2023 ©Alizé LJ

Esa iconografía y esos colores siguen presentes hasta el día de hoy, una escritura que resiste ante las dominaciones coloniales y el genocidio insistente presente en tierras indígenas.

Registro de la memoria

Maguey, algodón, plumas y otras fibras han sido materiales primordiales para la elaboración de las prendas. Cada material regala texturas únicas. Los antiguos pobladores cuidaban de estas plantas y/o animales que, al estar ligados con una actividad tan importante, pasaban a formar parte del mundo sagrado.

Actualmente en el estado de Chiapas, muchos de estos materiales han perdido tierra, ya que la introducción de nuevas plantas las ha desterrado. La creación de fincas cañeras ha causado problemas ambientales, económicos y políticos que se mantienen hasta el día de hoy. Como contraparte quizás, especies introducidas desde la Conquista se han integrado a la artesanía textil, como es el caso de oveja cuya lana se hila y se teje (**Figura 8**).

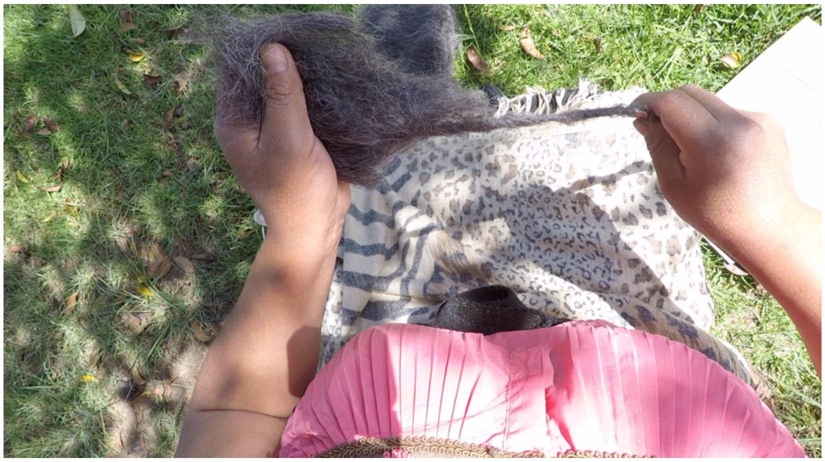

Figura 8: proceso de hilado de la lana en los Altos de Chiapas, 2023 ©Alizé LJ

Registro de la geografía

Una prenda tradicional perteneciente a un pueblo indígena puede revelar a qué tipo de entorno pertenece. En Chiapas, para el ojo contemplativo se podrá distinguir si corresponde a una tierra fría o caliente a través de sus colores y símbolos (**Figura 9**)

Azules, verdes, rojos, negros y grises, nos darán un contexto de montañas, cuevas y niebla. Los colores rosas, amarillos y morados nos compartirán su cálido aire, sus flores abundantes, ríos y lagos alimentando a toda la tierra.

Por otro lado, una simbología acompaña estas prendas. Cuenta historias únicas de tierras habitadas por sociedades comunitarias, símbolos específicos de un lugar, que adopta todo el pueblo. En tierra caliente, se suelen encontrar fauces de jaguar ornando el cuello de los huipiles. En tierra fría, se encuentran rumbos que remiten a las cuevas.

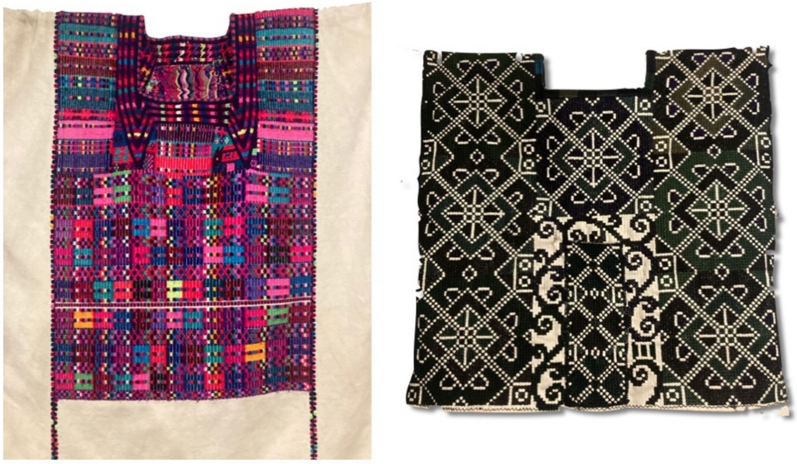

Figura 9: huipil de San Juan Cancuc con fauces de jaguar y colores de tierra caliente (izquierda); huipil de Zinacantán con cuevas y colores de tierra fría (derecha).

Esos símbolos nos hablan de lo que habita ese mundo, tanto en el terreno espiritual-mágico como en lo terrenal.

Registro narrativo

Los glifos y los símbolos que el hasta el día de hoy perduran en las prendas textiles son una narrativa específica de la cosmovisión antigua. Los hilos van formando líneas perfectas, que hacen alusión a formas cuadras, rombos y cruces. Los símbolos como los rumbos hablan tanto de los cuatro pilares de la tierra, como de sus varios niveles, que del útero materno y de la cueva como lugar de origen de los pueblos[10,11].

---

[10] Kolpakova, A. (2018). *Diseños mágicos: Análisis de los diseños con rombos en los huipiles mayas de Chiapas* (Segunda edición corregida y aumentada). Consejo Estatal para las Culturas y las Artes de Chiapas.

[11] López Austin, A., & López Luján, L. (2009). *Monte sagrado: Templo Mayor*. Instituto Nacional de Antropología e Historia Universidad Nacional Autónoma de México, Instituto de Investigaciones Antropológicas.

Las formas presentes en los textiles, al juntarse y dependiendo de su posición crean un lenguaje específico. Es de suponer que el color también sirve cómo un elemento para ayudar a esta narrativa de formas y pigmentos. Es por ello que sostenemos que es una escritura que ha resistido a los modernismos e implementaciones capitalistas.

Registro de uso

En las diferentes comunidades indígenas del estado de Chiapas, la ropa también ha servido para marcar diferencias sociales, ya que existen personas con cargos tradicionales relacionados con ceremonias específicas; petición de lluvia, mayordomos que guardan el rezo para el señor de la montaña, y mujeres que acompañan estas ceremonias. Guardan trajes ceremoniales hechos con material y tiempo diferentes, que solo ellos pueden utilizar en fechas específicas.

Es por ello que se defiende el uso de vestimenta en pueblos originarios. Más allá de una moda o una tendencia, está ligado a un mundo espiritual que explica la existencia del ser humano en la tierra.

Hilos de colores, formas, texturas y hasta el proceso de elaboración, no son coincidencia, una prenda puede revelar los secretos y misterios que susurran las cuevas antiguas.

ENTRADA LIBRE • **ABIERTO A TODO PÚBLICO** • **MÚSICA EN VIVO** • **COMIDA Y BEBIDAS**

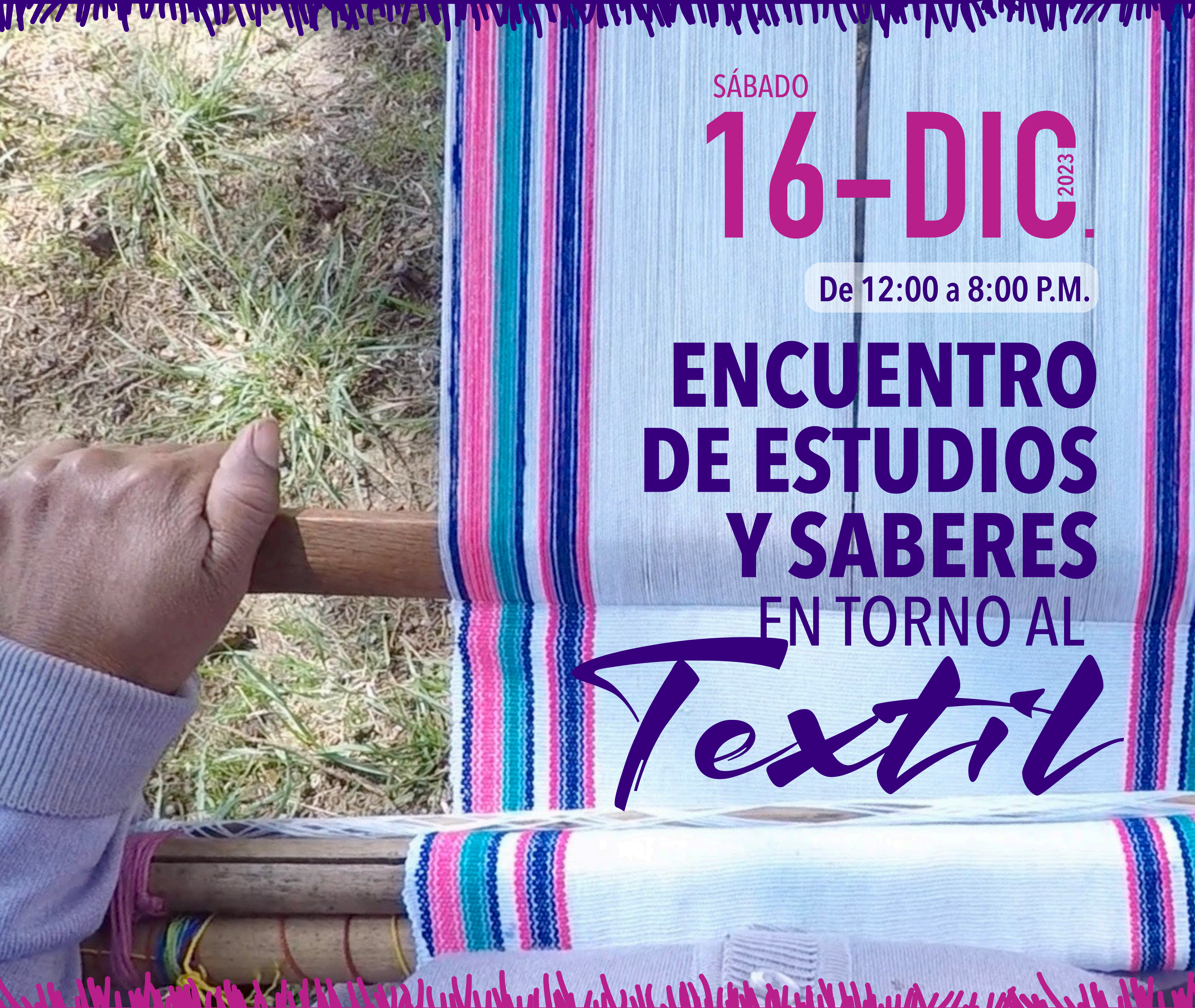

SÁBADO

16-DIC 2023.

De 12:00 a 8:00 P.M.

ENCUENTRO DE ESTUDIOS Y SABERES EN TORNO AL *Textil*

ACTIVIDADES:

- **Inauguración del espacio textil "Ixim"**

- **PLÁTICA:** "Trabajo de mujeres: la antigua producción de textiles".
  Berenice Jiménez González (UNAM–INAH)

- **EXHIBICIÓN FOTOGRÁFICA:** Artefactos del arte textil prehispánico.

- **PLÁTICA:** "El tejido de la historia: huesos y textiles ".
  Alizé Lacoste Jeanson & Rocio de la Cruz (UNAM–Tierra del Maíz)

- **Demostración de telar de cintura tradicional.**
  Adolfo Sántiz Pérez (Huixtán)
  Juana Sántiz Gómez (San Juan Cancuc)
  Matilde Arias Guzmán (Chenalhó)

- **PRESENTACIÓN DEL PROYECTO:** "MUK' TA LUCH": colectivo de bordado y de agroecología.

- **PRESENTACIÓN DEL PROYECTO:** "Dignificando nuestra historia": el arte de la animación comunitaria.
  Yasmin Samantha Ruíz Hernández (UNAM–Tierra del Maíz)

"LA TIERRA DEL MAÍZ" COLECTIVO CULTURAL

Calle Nicolás Ruíz 85-E. Barrio de Guadalupe.
San Cristóbal de Las Casas, Chiapas.
INFORMES: +52 967 165 78 93

MODIFICACIONES CORPORALES Y SÍMBOLOS

DENTRO DE LA COSMOVISIÓN MAYA

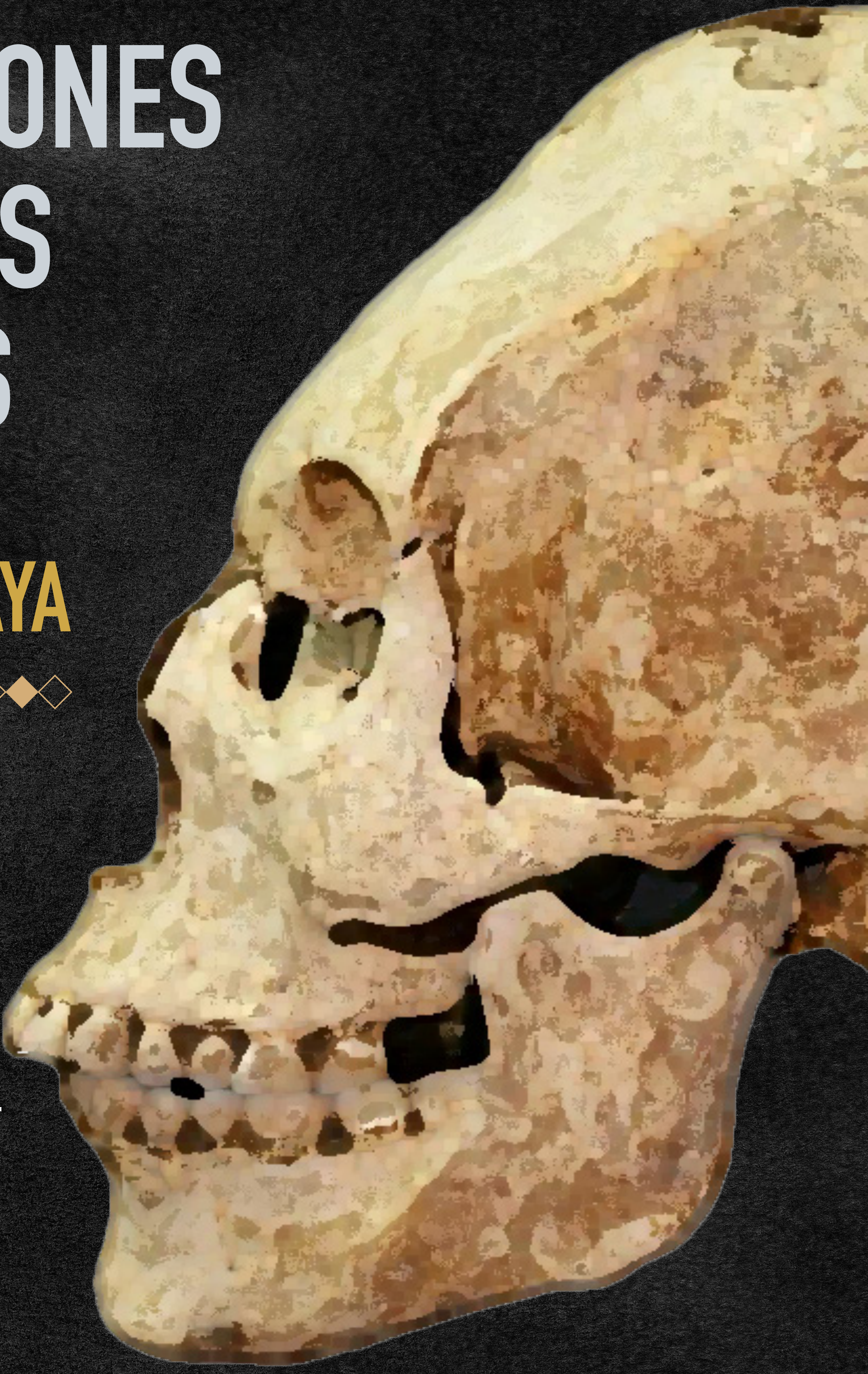

05 / OCT. / 2024

11:00 A.M. a 8:00 P.M.

CONFERENCIAS:

Moderación: **Heydi De la Cruz** (lic. en Antropología).

- "EL MODELO CEFÁLICO DE LOS MAYAS ANTIGUOS: APROXIMACIONES INTERPRETATIVAS".
  Alizé Lacoste Jeanson (Investigadora del Instituto de Investigaciones Antropológicas, UNAM).
- "SIMBOLOGIA EN EL ARTE TEXTIL TRADICIONAL DE LOS ALTOS DE CHIAPAS".
  Rocío De La Cruz (Lic. en Lengua y Literatura Hispanoamericana, Bordadora tradicional y promotora cultural de los pueblos originarios).
- "A PROPÓSITO DEL DESARRAIGO Y EL OLVIDO: UNA CRÍTICA EPISTEMOLÓGICA EN TIEMPOS NEOCOLONIALES".
  Daniel Alexis Faúndez Heras.
  (Lic. en Educación y Filosofía, originario de Wallmapu).
- "LA MODIFICACIÓN DENTAL EN MESOAMÉRICA Y SUS IMPLICACIONES SIMBÓLICAS".
  Josuhé Lozada Toledo (Investigador del INAH).
- "SIMBOLISMOS DEL ALTAR MAYA TSOTSIL: ENTRE HISTORIAS Y RITUALES".
  Laura Hernández Santíz (Lic. en Enseñanza del inglés, Maestra de tsotsil y fotógrafa independiente, originaria de Basheken, Chamula).

OTRAS ACTIVIDADES:

- VENTA Y DEGUSTACIÓN DE COMIDA TRADICIONAL DE SAN JUAN CHAMULA
- TATUAJES FLASH
  "Simbología textil de los Altos de Chiapas".
  Labryntho Estudio

LABRYNTHO ESTUDIO

LA TIERRA DEL MAÍZ
ixim
COLECTIVO CULTURAL

Nicolás Ruiz 85-E, Barrio de Guadalupe, San Cristóbal de Las Casas, Chiapas.
